# Putative Pharmacological Depression and Anxiety-Related Targets of Calcitriol Explored by Network Pharmacology and Molecular Docking

**DOI:** 10.3390/ph17070893

**Published:** 2024-07-05

**Authors:** Bruna R. Kouba, Glorister A. Altê, Ana Lúcia S. Rodrigues

**Affiliations:** Department of Biochemistry, Center of Biological Sciences, Federal University of Santa Catarina, Florianopolis 88037-000, SC, Brazil; rkba.bruna@gmail.com (B.R.K.); glorister.alte@ufsc.br (G.A.A.)

**Keywords:** vitamin D, antidepressant effects, anxiolytic effects, bioinformatic analysis

## Abstract

Depression and anxiety disorders, prevalent neuropsychiatric conditions that frequently coexist, limit psychosocial functioning and, consequently, the individual’s quality of life. Since the pharmacological treatment of these disorders has several limitations, the search for effective and secure antidepressant and anxiolytic compounds is welcome. Vitamin D has been shown to exhibit neuroprotective, antidepressant, and anxiolytic properties. Therefore, this study aimed to explore new molecular targets of calcitriol, the active form of vitamin D, through integrated bioinformatic analysis. Calcitriol targets were predicted in SwissTargetPrediction server (2019 version). The disease targets were collected by the GeneCards database searching the keywords “depression” and “anxiety”. Gene ontology (GO) and the Kyoto Encyclopedia of Genes and Genomes (KEGG) were used to analyze the intersections of targets. Network analyses were carried out using GeneMania server (2023 version) and Cytoscape (V. 3.9.1.) software. Molecular docking predicted the main targets of the network and Ligplot predicted the main intermolecular interactions. Our study showed that calcitriol may interact with multiple targets. The main targets found are the vitamin D receptor (VDR), histamine H3 receptor (H3R), endocannabinoid receptors 1 and 2 (CB1 and CB2), nuclear receptor NR1H3, patched-1 (PTCH1) protein, opioid receptor NOP, and phosphodiesterase enzymes PDE3A and PDE5A. Considering the role of these targets in the pathophysiology of depression and anxiety, our findings suggest novel putative mechanisms of action of vitamin D as well as new promising molecular targets whose role in these disorders deserves further investigation.

## 1. Introduction

Depression and anxiety disorders are common neuropsychiatric conditions, characterized by physiological alterations, which limit psychosocial functioning and, consequently, the individual’s quality of life [1,2]. Globally, it is estimated that more than 350 million people are affected by depression and 264 million by anxiety [3]. In addition, a significant increase has been reported in the prevalence of anxious and depressive symptoms since the coronavirus 2019 (COVID-19) pandemic [4].

Depression and anxiety often coexist, a fact that suggests the involvement of common pathophysiological factors to these disorders, including those related to gene alterations [5]. It is estimated that 85% of patients with depression also experience symptoms of anxiety. Likewise, a significant percentage of patients with anxiety disorders have comorbid depression [5,6]. Notably, comorbidity between these disorders is associated with greater symptom severity and a higher incidence of suicide [5]. Although selective serotonin reuptake inhibitors are the most widely prescribed pharmacological treatment in the clinic for the management of these disorders, these drugs have important limitations. For example, a high percentage of individuals exhibit only partial responses to treatment. In addition, the several side effects and the therapeutic window of 3–4 weeks corroborate patients’ low adherence to pharmacotherapy [1,7]. Therefore, there is a need to explore novel molecular mechanisms and pharmacological agents for the management of depression and anxiety disorders.

Vitamin D is a fat-soluble vitamin of vital importance that has been reported to exert neuroprotective effects [8]. Regarding the diagnostic limits for serum 25(OH)D levels, it has been established that deficient levels are values <50 nmol/L (20 ng/mL); insufficient values, between 50–80 nmol/L (20–32 ng/mL); adequate (considering sunny countries), 135–225 nmol/L (54–90 ng/mL); excess, >250 nmol/L (>100 ng/mL); and intoxication, >325 nmol/L (>130 ng/mL). Excessive intake of vitamin D through supplements can result in the accumulation of this vitamin in adipose tissue, inducing intoxication. Although cases of intoxication are rare, this condition is associated with adverse effects such as hypercalcemia and gastrointestinal discomfort [9,10].

**Figure 1:**
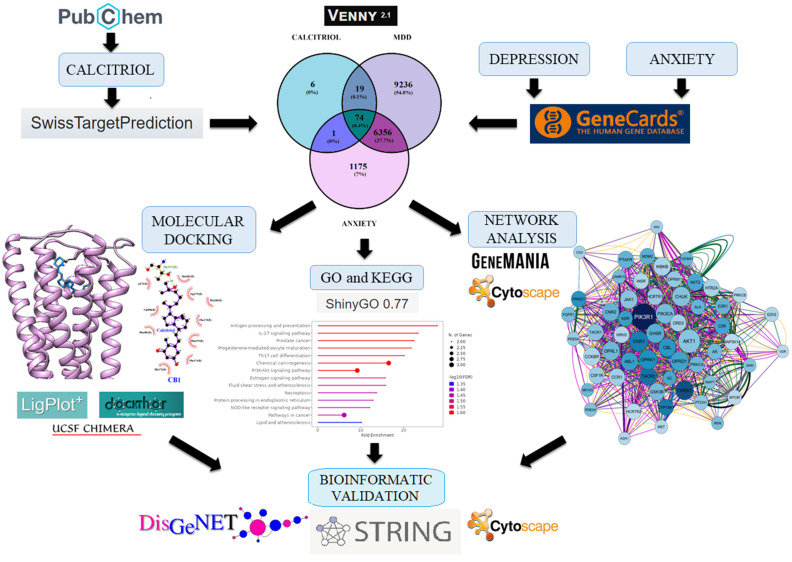
Schematic workflow showing the approach of this study. Genes modulated by calcitriol and genes related to depression and anxiety were searched in databases (Swiss Target Prediction and GeneCards). Subsequently, genes overlapping calcitriol, depression, and anxiety; genes overlapping calcitriol and depression; and genes overlapping calcitriol and anxiety were analyzed by Venn diagram, and then a pharmacological network using Genemania and Cytoscape software was built. Subsequently, gene ontology and KEGG analyses were carried out. Finally, molecular docking and interaction analyses of calcitriol with proteins were carried out using the Chimera, Dockthor, and LigPlot programs. To further validate the calcitriol targets, a STRING-DisGeNET network was constructed.

Robust evidence has emphasized the antidepressant and anxiolytic role of vitamin D [8,11,12]. In addition, deficient levels of vitamin D are associated with depression and anxiety symptoms in clinical studies [13,14,15]. This pleiotropic molecule performs various functions in the Central Nervous System (CNS) through its active metabolite, calcitriol. It is well-established in the literature that calcitriol’s genomic properties depend on its interaction with its receptor, the VDR (vitamin D receptor) [16,17]. However, non-genomic mechanisms related to the interaction between calcitriol and other proteins have been poorly explored. It is known that this metabolite can interact with PDIA3 (protein disulfide isomerase family A member 3) present in the plasma membrane, thus regulating calcium influx and intracellular pathways [16]. However, current studies are limited to evaluating the effects of calcitriol mediated by the VDR and PDIA3. Therefore, this study aimed to explore novel molecular targets of calcitriol that may underlie its antidepressant and anxiolytic properties through analyses involving networks of genes with pharmacological potential, gene ontology enrichment, and molecular docking. A flowchart of the study is presented in Figure 1.

## 2. Results

### 2.1. Identification and Gene Overlap between Potential Therapeutic Targets of Calcitriol and Genes Related to Depression and/or Anxiety

Analysis of the data obtained by the SwissTargetPrediction software allowed for the identification of 100 potential therapeutic targets modulated by calcitriol. Regarding genes related to depression and anxiety, the GeneCards database indicated a total of 15,686 and 7606 genes, respectively.

**Figure 2:**
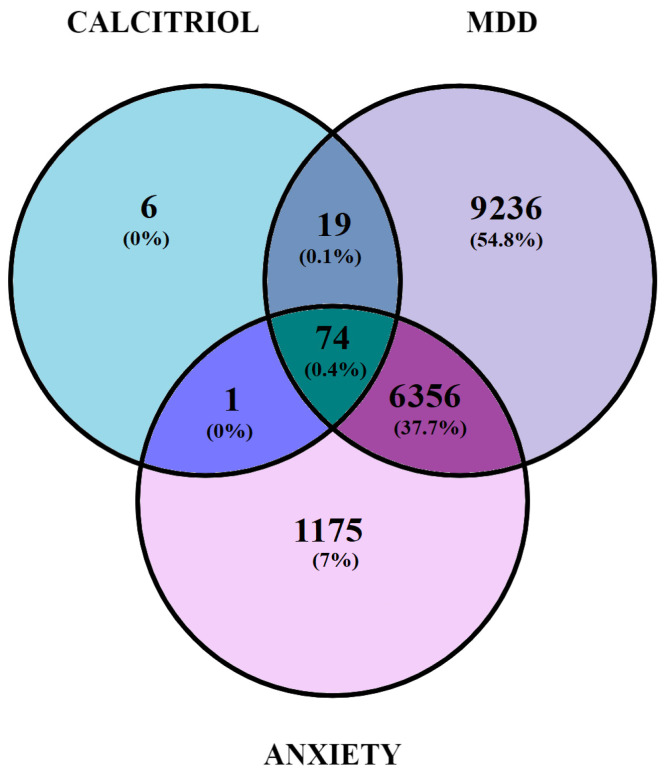
Venn diagram illustrating the potential target genes related to depression, anxiety, and calcitriol.

The Venn diagram (Figure 2) shows 74 genes overlapping calcitriol, depression, and anxiety (Table 1), 19 genes overlapping calcitriol and Major Depressive Disorder (MDD) (*PTPN1*; *GSK3A*; *GPBAR1*; *DGAT1*; *EPHB4*; *NPC1L1*; *JAK3*; *PIM1*; *ADAMTS5*; *ADAMTS4*; *SMO*; *EBP*; *AKT3*; *WEE1*; *S1PR3*; *S1PR1*; *MMP13*; *STAT6*; and *TTK*), and a single gene overlapping anxiety and calcitriol (*aurora kinase B*).

#### 2.1.1. Elaboration of Pharmacological Networks of Calcitriol, Depression, and Anxiety-Overlapping Genes

**Figure 3:**
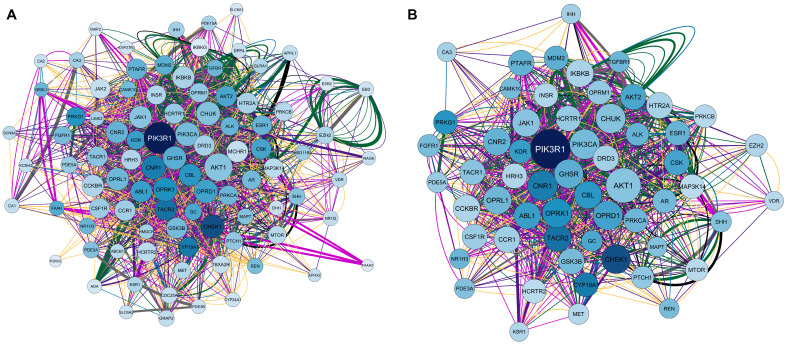
Network interactions among calcitriol, anxiety, and depression targets. (**A**) Complete network composed of 94 nodes. (**B**) Filtered network containing 59 most central and connected target genes shared among calcitriol, anxiety, and depression. The network was based on the degree center (DC) ≥ 21, betweenness centrality (BC) range of 0.004–0.046, and closeness centrality (CC) range of 0.477–0.780. The largest node indicates a higher degree in the network. The color of the edges refers to the types of interactions: physical interactions (green), co-expression (yellow), shared protein domains (pink), co-locations (blue), predictions (gray), genetic interactions (purple), and metabolic pathways (black).

Using the Homo sapiens filter, the overlapping genes for calcitriol, depression, and anxiety were searched in the Genemania software. Subsequently, the obtained network was examined in Cytoscape V. 3.9.1. software (The Cytoscape Consortium, San Diego, CA, USA). Figure 3A shows the network, which is composed of 94 nodes, 1692 connections (number of edges), and a clustering coefficient of 0.379. Notably, of the 94 nodes obtained, 20 genes were recovered: *IHH, KSR1, GRAP2, PRKCA, CBL, PTCH1, DHH, OPRL1, CAMK1G, PDE5A, CA3, EED, IKBKG, NAGK, PIK3R1, QRSL1, MAPT, ADA, APPL1,* and *FAAH2*. In the network constructed, a larger node size indicates that a given protein has a greater number of connections. In addition, the darker the color of the node, the greater the centrality of intermediation. Therefore, the *PIK3R1* gene, which encodes phosphoinositide-3-kinase regulatory subunit 1, stands out in this network. Regarding the connections, of the 1692 edges obtained, 31.30% were physical interactions (green), 25.83% were co-expressed (yellow), 16.81% were genetic interactions (purple), 8.84% were co-localized (blue), 8.11% represented shared protein domains (pink), 7.63% were predictions (gray), and 1.48% were metabolic pathways (black).

Finally, considering a degree ≥21, betweenness centrality between 0.004 and 0.046, and closeness centrality between 0.477 and 0.780, the network was filtered. As a result, 59 genes with the highest numbers of connections (degree ≥ 21) and the shortest paths (betweenness centrality) in the network were obtained. The filtered network is illustrated in Figure 3B, and the 59 genes with their respective codified proteins are listed in Table 2.

#### 2.1.2. Elaboration of Pharmacological Networks of Genes Overlapping for Calcitriol and Depression

**Figure 4:**
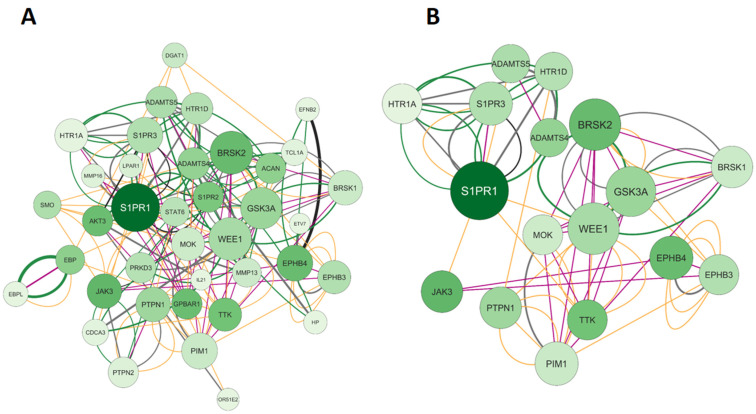
Network interactions between calcitriol and depression targets. (**A**) Complete network composed of 39 nodes. (**B**) Filtered network containing 17 genes was obtained using the parameters: degree center (DC) > 9, betweenness centrality (BC) range of 2.000–0.335, and closeness centrality (CC) range of 0.300–6.000. The largest node indicates a higher degree in the network. The color of the edges refers to the types of interactions: physical interactions (green), co-expression (yellow), shared protein domains (pink), co-locations (blue), predictions (gray), genetic interactions (purple), and metabolic pathways (black).

A Venn diagram revealed 19 genes that are shared by calcitriol and depression. Based on this result, we constructed and analyzed the network illustrated in Figure 4A. The network consists of 39 nodes, 158 connections, and a clustering coefficient of 0.305. The 39 nodes obtained are related to 19 shared genes plus 20 genes retrieved in the network: *ETV7, IL-21, HTR1A, HP, EFNB2, MMP16, EBPL, PRKD3, BRSK1, HTR1D, CDCA3, ACAN, BRSK2, OR51E2, S1PR2, MOK, LPAR1, TCL1A, EPHB3,* and *PTPN2.* Furthermore, the most important gene in this network is S1PR1, which encodes sphingosine-1-phosphate receptor 1. Regarding the connections, of the 158 edges, 38.96% were predictions (gray), 34.39% were co-expressed (yellow), 13.59% were metabolic pathways (black), 9.16% were shared protein domains (pink), and 3.90% were physical interactions (green). Subsequently, the network was filtered based on the following topological data: degree > 9, betweenness centrality between 2.000 and 0.335, and closeness centrality between 0.300 and 6.000. After applying the filters, we obtained a network with 17 genes, as shown in Figure 4B. The genes and their respective proteins include *HTR1A (5-hydroxytryptamine receptor 1A), S1PR1 (sphingosine-1-phosphate receptor 1), S1PR3 (sphingosine-1-phosphate receptor 3), ADAMTS5 (ADAM metallopeptidase with thrombospondin type 1 motif 5), HTR1D (5-hydroxytryptamine receptor 1D), ADAMTS4 (ADAM metallopeptidase with thrombospondin type 1 motif 4), BRSK2 (BR serine/threonine kinase 2), JAK3 (Janus kinase 3), PTPN1 (protein tyrosine phosphatase non-receptor type 1), MOK (MOK protein kinase), WEE1 (WEE1 G2 checkpoint kinase), GSK3A (glycogen synthase kinase 3 alpha), PIM1 (Pim-1 proto-oncogene, serine/threonine kinase), TTK (TTK protein kinase), EPHB4 (EPH receptor B4), EPHB3 (EPH receptor B3),* and *BRSK2 (BR serine/threonine kinase 2)*.

#### 2.1.3. Elaboration of Pharmacological Networks of Genes Overlapping Calcitriol and Anxiety 

**Figure 5:**
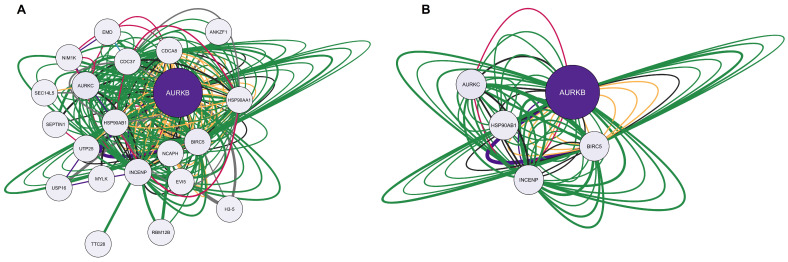
Network interactions between calcitriol and anxiety targets. (**A**) Complete network containing 21 nodes. (**B**) Filtered network composed of 5 nodes was obtained using the parameters: network based on the degree center (DC) > 5, betweenness centrality (BC) range of 0.034–0.900, and closeness centrality (CC) range of 0.528–1.000. The largest node indicates a higher degree in the network. The color of the edges refers to the types of interactions: physical interactions (green), co-expression (yellow), shared protein domains (pink), co-locations (blue), predictions (gray), genetic interactions (purple), and metabolic pathways (black).

Figure 5A represents the network formed by the only gene overlapping calcitriol and anxiety, AURKB. The network obtained consists of 21 nodes, 240 connections, and a clustering coefficient of 0.570. In addition to *AURKB*, the network recovered 20 other genes, including *ANKZF1, H3-5, RBM12B, TTC28, EVI5, SEPTIN1, USP16, MYLK, NIM1K, EMD, AURKC, SEC14L5, UTP25, CDC37, HSP90AB1, HSP90AA1, INCENP, CDCA8, BIRC5,* and *NCAPH*. Regarding connections, of the 240 edges, the majority were physical interactions at 77.64% (green), 8.01% were co-expressed (yellow), 5.37% were predictions (gray), 3.63% were co-localized (blue), 2.87% were genetic interactions (purple), 1.88% were metabolic pathways (black), and 0.60% were shared protein domains (pink). After applying the filters degree >5, betweenness centrality between 0.034 and 0.900, and closeness centrality between 0.528 and 1.000, we obtained the main five genes of the network: *AURKB (aurora kinase B), AURKC (aurora kinase C), HSP90AB1 (heat shock protein 90 alpha family class B member 1), INCENP (inner centromere protein),* and *BIRC5 (baculoviral IAP repeat containing 5)*. The filtered network is illustrated in Figure 5B.

### 2.2. Kyoto Encyclopedia of Genes and Genomes (KEGG) and Gene Ontology (GO) Enrichment Analysis of Genes Overlapping Calcitriol, Depression and Anxiety 

**Figure 6:**
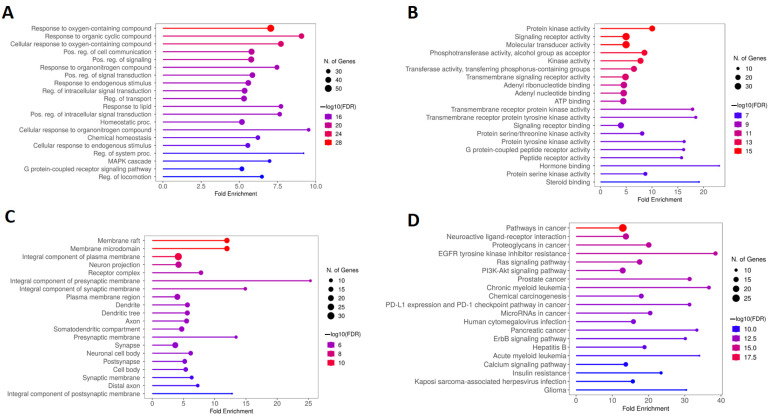
GO and KEGG enrichment analysis of hub targets among genes overlapping calcitriol, depression, and anxiety. The GO enrichment analysis obtained 20 main terms related to (**A**) BP, (**B**) MF, and (**C**) CC. (**D**) The top 20 pathways were obtained by KEGG analysis. Abbreviations: BP, biological process; MF, molecular function; CC, cell composition.

KEGG and GO analyses were conducted to determine the biological functions and molecular pathways related to calcitriol in depression and anxiety. Figure 6A illustrates the five main terms obtained from the biological process (BP) analysis of GO, which include response to oxygen-containing compound, response to organic cyclic compound, cellular response to oxygen-containing compound, positive regulation of cell communication, and positive regulation of signaling. In addition, based on the enrichment of folds, the functions of cellular response to organonitrogen compounds, and regulation of the system process were highlighted. The molecular function (MF) results, shown in Figure 6B, describe the main five terms which were protein kinase activity; signaling receptor activity; molecular transducer activity; phosphotransferase activity, alcohol group as acceptor; and kinase activity. Moreover, hormone binding, steroid binding, and transmembrane receptor protein tyrosine kinase had a high enrichment in MF. Figure 6C shows the five main cellular components (CC) which included the membrane raft, membrane microdomain, integral component of plasma membrane, neuron projection, and receptor complex. By analyzing the CC, it was also possible to verify that these genes are important for composing the presynaptic and synaptic membranes. Finally, the five main pathways obtained by KEGG analysis were pathways in cancer, proteoglycans in cancer, neuroactive ligand–receptor interaction, EGFR (epidermal growth factor receptor) tyrosine kinase inhibitor resistance, and Ras signaling pathway (Figure 6D).

### 2.3. KEGG and GO Enrichment Analysis of Genes Overlapping Calcitriol and Depression 

**Figure 7:**
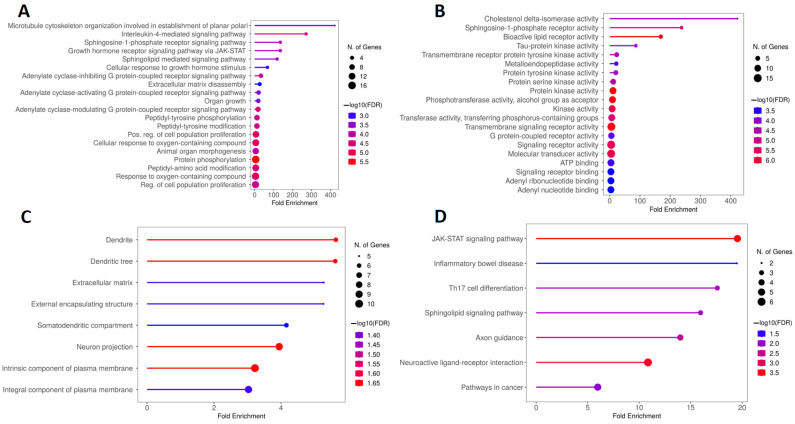
GO and KEGG enrichment analysis of hub targets among genes overlapping calcitriol and depression. The GO enrichment analysis obtained 20 main terms related to (**A**) BP, (**B**) MF, and (**C**) CC. (**D**) The top 20 pathways were obtained by KEGG analysis. Abbreviations: BP, biological process; MF, molecular function; CC, cell composition.

Figure 7A is composed of the five main BPs obtained from the GO analysis, which include microtubule cytoskeleton organization involved in the establishment of planar polari and the signaling pathways mediated by interleukin-4, the sphingosine-1-phosphate receptor; the growth hormone receptor via JAK/STAT (Janus kinase/signal transducer and activator of transcription); and sphingolipids. Regarding the MF, Figure 7B illustrates that the main terms obtained were cholestenol delta-isomerase activity, sphingosine-1-phosphate receptor activity, bioactive lipid receptor activity, tau-protein kinase activity, and transmembrane receptor protein tyrosine kinase activity. Figure 7C, in turn, shows that the proteins encoded by the genes obtained from the calcitriol and depression network were preferentially located in the dendrite, dendritic tree, extracellular matrix, external encapsulating structure, and somatodendritic compartment. Finally, Figure 7D illustrates the five main terms obtained by KEGG analysis: the JAK-STAT signaling pathway, inflammatory bowel disease, Th17 cell differentiation, sphingolipid signaling pathway, and axon guidance.

### 2.4. KEGG and GO Enrichment Analysis of Genes Overlapping Calcitriol and Anxiety 

**Figure 8:**
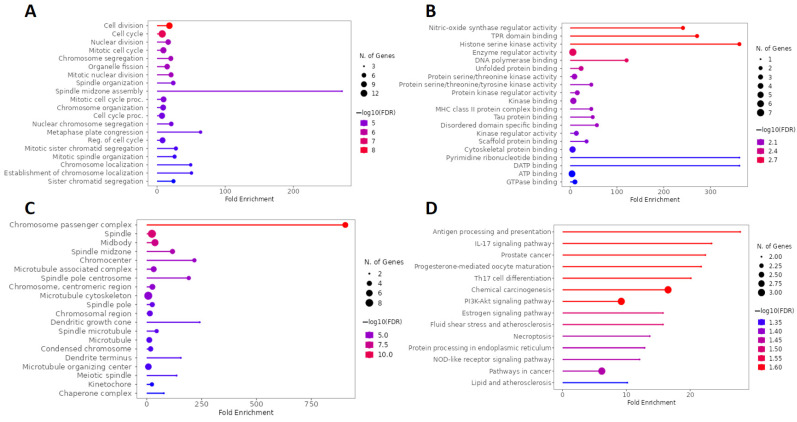
GO and KEGG enrichment analysis of hub targets among genes overlapping calcitriol and anxiety. The GO enrichment analysis obtained 20 main terms related to (**A**) BP, (**B**) MF, and (**C**) CC. (**D**) The top 20 pathways were obtained by KEGG analysis. Abbreviations: BP, biological process; MF, molecular function; CC, cell composition.

The main BPs involved in the analysis of the network formed by genes overlapping calcitriol and anxiety are related to cell division mechanisms such as the cell cycle, nuclear division, mitotic cell division, and chromosome segregation. In addition, regarding fold enrichment, the spindle midzone assembly stood out (Figure 8A). Figure 8B shows the five main terms related to MF, which include nitric-oxide synthase regulator activity, TPR (tetratricopeptide repeat) domain binding, histone serine kinase activity, enzyme regulator activity, and DNA polymerase binding. In addition, pyrimidine ribonucleotide binding and dATP (deoxyadenosine triphosphate) binding showed a high enrichment in MF. Regarding the CCs, the top five terms found were chromosome passenger complex, spindle, midbody, spindle midzone, and chromocenter (Figure 8C). Finally, Figure 8D illustrates the main pathways obtained by KEGG analysis, which include antigen processing and presentation, IL-17 signaling pathway, prostate cancer, progesterone-mediated oocyte maturation, and Th17 cell differentiation.

### 2.5. Molecular Docking Simulations

The results of the docking analyses revealed that calcitriol has a polypharmacological profile, with the potential to interact with various molecular targets. We selected those targets whose affinity values were equal to or less than −11 kcal/mol. Another criterion for the selection was that the affinity predictions obtained from the positive control with well-established ligands were lower than those obtained with calcitriol. The vitamin D receptor (VDR), one of the main pharmacological targets of calcitriol, was the top target in molecular docking (−12.116 kcal/mol). The following pharmacological targets of calcitriol were HRH3 (−12.077 kcal/mol), NR1H3 (−11.927 kcal/mol), CNR1 (−11.810 kcal/mol), PTCH1 (−11.570 kcal/mol), and CNR2 (−11.333 kcal/mol). Furthermore, PDE3A, PDE5A, and OPRL1 were also considered relevant targets because they showed more affinity for calcitriol than their respective positive controls (Table 3). Molecular docking parameters are available in the Supplementary Materials.

**Figure 9:**
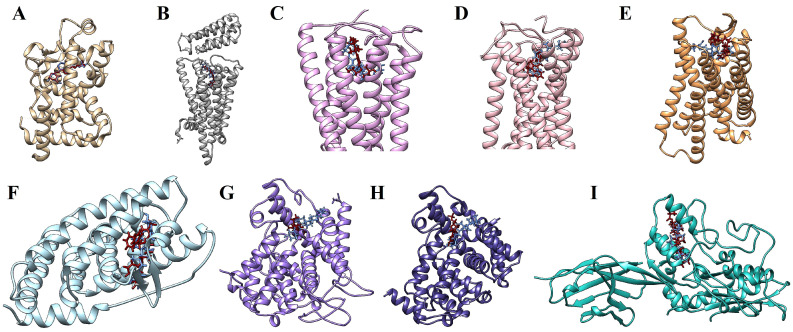
Molecular docking simulations in which calcitriol is colored in blue and the positive controls are colored in dark red. (**A**) VDR and calcitriol interacting in VDX binding site. (**B**) H3R and calcitriol in the 1IB binding site. (**C**) CB1 and calcitriol in the 9GF binding site. (**D**) CB2 and calcitriol in the WI5 binding site. (**E**) OPRL1/NOP and calcitriol in the DGV binding site. (**F**) NR1H3/LXR-α and calcitriol in the 965 binding site. (**G**) PDE3A and calcitriol in the X5M binding site. (**H**) PDE5A and calcitriol in the 5GP binding site. (**I**) PTCH1 and calcitriol in the Y01 binding site.

**Figure 10:**
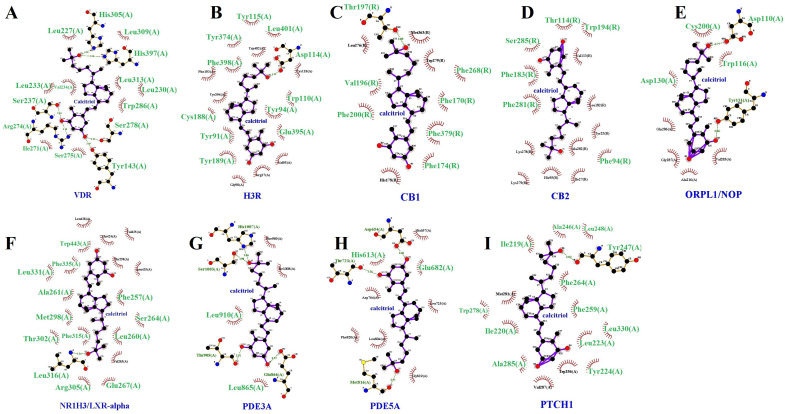
The Ligplot + diagrams of the molecular docking results between the calcitriol and protein targets. Representative pose of calcitriol complexed with protein targets in which hydrogen bonds are represented as green dotted lines and hydrophobic interactions in red spoked arcs. Two-dimensional representation of protein–calcitriol interactions for the VDR (**A**), H3R (**B**), CB1 (**C**), CB2 (**D**), OPRL1/NOP (**E**), NR1H3/LXR-α (**F**), PDE3A (**G**), PDE5A (**H**), and PTCH1 (**I**). The amino acid residues highlighted (larger size and green color) are those shared between calcitriol and the respective positive controls.

Figure 9 illustrates the molecular docking simulations, indicating the binding sites between calcitriol and the target proteins. Ligplot was used to study the protein–calcitriol interactions. Overall, calcitriol has many hydrophobic interactions with the different targets. Figure 10 shows the hydrogen bonds and the hydrophobic interactions between calcitriol and our selected targets.

Besides the targets shown in Table 3, the molecular docking scores also suggest that calcitriol may interact with other targets, as represented in Table 4 and Table 5.

### 2.6. Bioinformatic Validation

**Figure 11:**
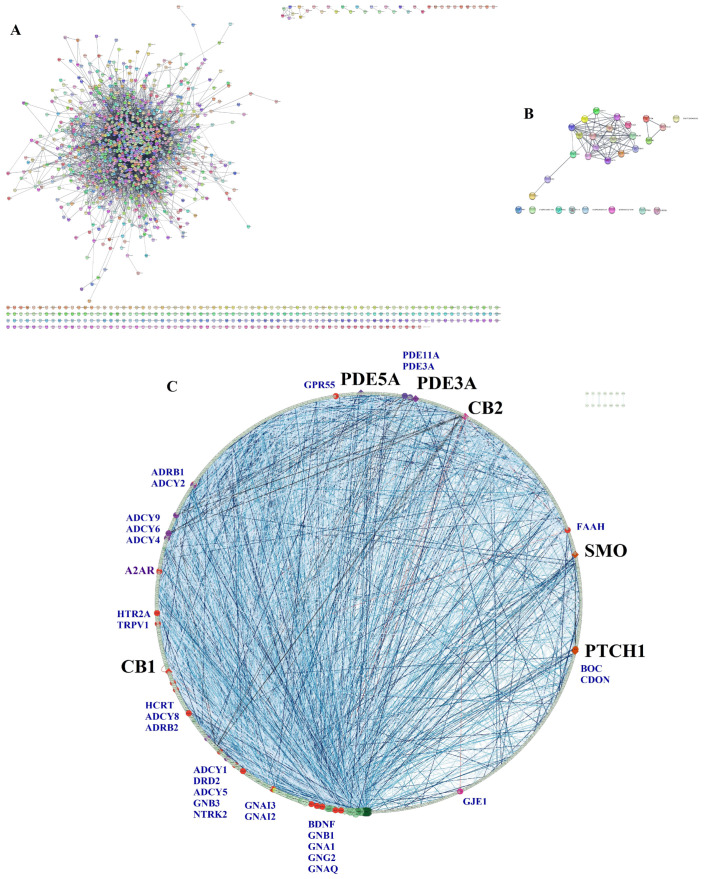
Bioinformatic validation networks. (**A**) Mental Depression PPI Network obtained in STRING and DisGENET. (**B**) Calcitriol top 10 targets PPI Network obtained in STRING. (**C**) PPI merged network constructed in STRING and Cytoscape of depression-related targets searched in DisGENET and 10 most relevant calcitriol potential targets obtained by molecular docking. Merged network is represented in circular layout by degree; the colors of nodes in green represent the highest betweenness centrality, the size of the node is represented by degree values, and the closeness centrality is represented by transparency. The edges were colored by experimentally determined interaction, with the intensity of the blue linked to the highest confidence value in this parameter. Of the top 10 promising targets of calcitriol in molecular docking (VDR, CB1, PDE5A, PDE3A, CB2, SMO, PTCH1, OPRL1, HRH3, and NR1H3), we were able to find 6 of these targets (CB1, PDE5A, PDE3A, CB2, SMO, and PTCH1 represented in black label) in the DisGeNET depression network. The proteins that interact with the target proteins of calcitriol are shown in blue and in smaller size.

A list of 1478 genes related to mental depression was obtained from DisGENET. From this list, a large PPI network was created in STRING with the following characteristics: 1398 nodes/proteins, 10,893 edges, an average node degree of 15.6, and an average clustering coefficient of 0.404 (Figure 11A). The top 10 calcitriol targets selected by the molecular docking values (VDR, CB1, PDE5A, PDE3A, CB2, SMO, PTCH1, OPRL1, HRH3, and NR1H3) were used to make another PPI network in STRING. The network of calcitriol pharmacological targets in STRING had the following characteristics: 30 nodes, 120 connections, an average node degree of 8, and an average clustering coefficient of 0.64 (Figure 11B). We merged the two networks in Cytoscape, which resulted in a final network with 1241 nodes, 10,991 edges, and a clustering coefficient of 0.383 (Figure 11C). The edges were colored according to the experimental evidence; the darker, the stronger the evidence. In this network, six calcitriol targets are present: CB1, PDE5A, PDE3A, CB2, SMO, and PTCH1. The topological parameters of these nodes in this network are presented in Table 6. In this network composed by 1241 proteins (organized by descending degree value), the positions of CB1, PDE5A, PDE3A, CB2, SMO, and PTCH1 are 241, 635, 690, 714, 949, and 1020, respectively. The calcitriol target proteins establish connections with other proteins highlighted in blue in the network (Figure 11C). Among these proteins are brain-derived neurotrophic factor (BDNF), components of the endocannabinoid system (FAAH, TRPV1, and GPR55), the adenosine receptor A2A (A2AR), the dopamine D2 receptor (DRD2), and the serotonin receptor HTR2A.

## 3. Discussion

Several lines of evidence report that deficient serum levels of vitamin D are related to depression and anxiety symptoms in clinical studies [13,14,15], and its supplementation is capable of exerting effects similar to those of antidepressants and anxiolytics in animal models [12,18,19,20,21]. Based on the benefits of this vitamin in either depression or anxiety disorders, we explored the possible molecular targets of calcitriol, the active form of vitamin D, in these disorders, using network pharmacology and molecular docking simulations. In the present study, we obtained three pharmacological networks: genes overlapping calcitriol, depression, and anxiety; genes overlapping calcitriol, and depression; and genes overlapping calcitriol, and anxiety. Considering the multiple targets obtained in network pharmacology, we evaluated the main interactions between the targets and calcitriol that showed the highest binding energy (values > −11) (Table 3). In addition, we highlighted some molecular targets in which the binding energy of calcitriol was higher than the one of the selected ligand. Thus, the main targets obtained were nuclear vitamin D receptor (VDR), nuclear receptor subfamily 1 group H member 3 (NR1H3), histamine H3 receptor (HRH3), protein patched homolog 1 (PTCH1), endocannabinoid receptors (CNR1 and CNR2), opioid-related nociceptin receptor 1 (OPRL1), and phosphodiesterases (PDE5A and PDE3A). Noteworthy, all these targets modulated by calcitriol are related to depression and anxiety. In addition, the STRING-DisGeNET network reinforced the relevance of these targets.

The vitamin D receptor was shown to be a target for calcitriol by the redocking analysis (−12.116 kcal/mol), as expected. The VDR is a member of the nuclear receptor superfamily of transcription factors and is the main member responsible for vitamin D genomic actions [16]. Calcitriol interacts directly with the VDR by facilitating the formation of a complex with the retinoic acid X receptor (RXR). Once formed, this complex is capable of interacting with vitamin response elements (VDREs), modulating the expression of specific genes [17,22]. Calcitriol is also able to interact directly with VDRs expressed in the plasma membrane, which can induce an influx of calcium and, consequently, the activation of signaling pathways mediated by kinases and phospholipases [16,23]. Besides this mechanism, the binding of calcitriol to the membrane VDR favors the interaction of this metabolite with other transcription factors, such as signal transducer and activator of transcription 3 (STAT3), nuclear factor kappa-B (NF-κB), and nuclear factor erythroid 2-related factor 2 (Nrf2) [24]. This gene modulation results in an increased expression of neurotransmitters [25,26], neurotrophic factors [27,28], and antioxidant enzymes [29]. It also leads to a decreased expression of pro-inflammatory mediators [30], contributing to the maintenance of cerebral homeostasis [16]. In this context, the polymorphisms of this receptor are reported to be associated with the development of psychiatric disorders [31,32,33]. In animal models, this receptor has been shown to play an essential role against depression-like and anxiety-like behaviors [34,35,36]. Reinforcing the anxiolytic role of VDR activation, a study conducted on VDR knockout mice found that the ablation of this receptor induced anxiety-like behavior [34]. Interestingly, an increase in the hippocampal VDR expression was observed in rats that exhibited stress-induced depressive-like behavior, likely due to a compensatory mechanism [35].

The histaminergic neurotransmission system regulates functions such as learning, memory, the sleep–wake cycle, and appetite regulation [37]. Dysfunctions in this system are related to psychiatric disorders such as anxiety and depression [38]. Histamine exerts its effects by interacting with four different G protein-coupled histamine receptors (HRs): H1R, H2R, H3R, and H4R. These receptors are responsible for distinct functions, with H1R involved in allergic responses, H2R involved in gastric acid secretion, H3R modulating neurotransmission systems, and H4R mediating inflammatory responses [38]. In our analyses, the HRH3 gene was found to be an important node in the calcitriol, depression and anxiety-overlapping network. Accordingly, gene ontology analyses (molecular function) indicated receptor signaling activity and G protein-coupled receptors as important pathways.

H3R is a pre-synaptic inhibitory autoreceptor expressed in GABAergic, glutamatergic, serotoninergic, noradrenergic, and cholinergic neurons, mainly in the cerebral cortex, thalamus, and hypothalamus [38]. In addition, according to the cellular composition, components of presynaptic membranes and the synapse itself were enriched. The molecular docking values revealed a high affinity of calcitriol for H3R (−12.007 kcal/mol), and this value was very close to the affinity of calcitriol for VDR (−12.116 kcal/mol). Furthermore, according to molecular docking analyses, calcitriol has a higher affinity than the positive control H3R antagonist PF03654746 (−10.509 kcal/mol) for this receptor. Several studies have demonstrated the neuroprotective, cognitive enhancement, and antidepressant effects of H3R antagonists or inverse agonists [39,40,41,42]. Some studies have also shown anxiolytic effects of H3R antagonists/or H3R genetic knockout [40,42,43], although other studies have shown anxiogenic effects of H3R antagonists [44]. H3R has been predicted as a promising target of calcitriol, responsible at least in part for its antidepressant and anxiolytic effects, a finding that deserves future studies.

Our study indicates that LXR is also a promising target for investigating the antidepressant and anti-inflammatory effects of vitamin D. The nuclear receptor subfamily 1 group H member 3 (NR1H3) gene encodes for the nuclear receptor protein NR1H3, also called Liver X receptor α (LXR-α) [45]. NR1H3 is a gene highly expressed in M(hb) macrophages in MDD patients [45] and in the plasma of rodents subjected to chronic unpredictable mild stress [46]. LXR nuclear receptors regulate lipid and cholesterol metabolism and inhibit the expression of pro-inflammatory genes in immune cells. These receptors have a ligand-binding domain (LBD) where phytosterols and cholesterol-like molecules act as agonists causing conformational changes in the molecule that change its affinity for repressor and coactivator molecules, facilitating heterodimerization with retinoid X nuclear receptors (RXR) to activate transcription [47]. According to our predictions, calcitriol has high activity for NR1H3 at this site (−11.927 kcal/mol), an affinity that is relatively close to that of the positive control GW-3965 (−13.950 kcal/mol). In agreement, vitamin D3 and its metabolites are able to interact with LXR receptors [48]. The agonist GW3965 that acts on both LXR-α and LXR-β elicited antidepressant-like effects, enhanced hippocampal neurogenesis, and improved myelination [49,50]. The LXR-α agonist T0901317 exerts anti-inflammatory effects by inhibiting NF-κB and the Nod-like receptor pyrin containing protein 3 (NLRP3), two well-known pro-inflammatory signaling pathways [51]. LXR is also involved in anxiety, but the role of the. LXR-α isoform in this regard has been poorly established. Ephytoxin, a molecule that has an anxiolytic effect, was able to reverse the expression profile of genes upregulated in obese animals, including the LXR-α gene [52]. The endocannabinoid system plays a significant role in regulating body homeostasis, being involved in the regulation of mood, appetite, memory, cognition, locomotor activity, and immune responses [53,54,55,56]. The CNR1 and CNR2 genes, enriched in our network analyses, code, respectively, for endocannabinoid receptors 1 (CB1) and 2 (CB2), which are inhibitory G-protein-coupled receptors [53,57]. The molecular docking results revealed that calcitriol has an affinity for CB1 (−11.810 kcal/mol) and CB2 (−11.333 kcal/mol), similar to the CB1 agonist CP55940 (−11.133 kcal/mol) and the CB1 and CB2 agonist WIN 55212-2 (−11.502 kcal/mol). The beneficial effects of vitamin D on depression and anxiety may be related to the potential interaction of this molecule with endocannabinoid receptors, and this possibility deserves future studies.

CB1s are presynaptic receptors expressed in the cerebellum and cortex [58] in oligodendrocytes and neurons [59] and are responsible for mediating the inhibition of neurotransmitter release in GABAergic and glutamatergic neurons [60]. Several molecules interact with CB1 receptors, such as ∆9-tetrahydrocannabinol (THC), THC analogues (such as dronabinol and nabilone), other synthetic molecules (HU-120, CP55940, and WIN55212), as well as endogenous ligands anandamide and 2-arachidonoylglycerol [61,62]. Several studies show that CB1 agonists have antidepressant [54,62,63], anxiolytic [62,64,65], and psychotropic [57,66] effects. On the other hand, CB1 antagonists or CB1 genetic knockout/knockdown show depressive-like and anxiolytic-like effects in animal models of stress [64,67]. Cannabidiol, a molecule that binds to several targets including CB1 and is classified as an inverse agonist or negative allosteric modulator of this receptor, exhibits anxiolytic effects [56,68,69]. Rimonabant, a selective CB1 antagonist, was developed for the treatment of obesity and metabolic syndrome due to its effects on weight loss, appetite suppression, and loss of visceral fat but was removed from the market due to serious side effects (depression, anxiety, and suicidality) in patients with a history of depressive symptoms [70,71].

CB2 is more restricted to organs of the immune system such as the spleen, thymus, and bone marrow and is expressed in oligodendrocytes, B lymphocytes, NK cells, and microglial cells [56,57,58,60,72]. There are several studies indicating the importance of CB2 modulation in depression and anxiety. Transgenic mice that overexpress CB2 are resilient to acute and chronic stimuli that generally trigger anxiety and depression [72]. On the other hand, the lack of CB2 or the pharmacological blockade of CB2 by antagonists or inverse agonists are associated with vulnerability to stress and the development of anxiety and depressive-like behaviors [73,74]. Some studies point to the antidepressant and anxiolytic effects of various cannabinoid compounds and synthetic agonists [72]. CB2 activation in microglia induces an anti-inflammatory phenotype in these cells [72]. Evidence suggests that activation of CB2 decreases the anxiety-like phenotype induced by chronic exposure to alcohol by inhibiting the NLRP3-driven pathway in microglia [75]. Reinforcing the possible relationship between vitamin D and the endocannabinoid system, vitamin D-deficient mice showed reduced spinal CB1 expression levels and increased CB2 expression levels [76].

The sonic hedgehog (SHH) signaling pathway is very active during the embryonic period of invertebrates and vertebrates, being important in the formation of the neural tube [77,78]. In adults, this pathway is related to wound healing and stem cells, such as neural stem cells. Dysregulation of this pathway is linked to several types of cancer [77] but also to diseases of the central nervous system, including depression [79]. PTCH1 is a sonic hedgehog (SHH) receptor protein and acts as a tumor suppressor gene, which suppresses the smoothened (SMO) protein. When SHH binds to PTCH1, SMO is released and signals cell proliferation. In rodents subjected to chronic unpredictable stress, this pathway is deregulated in the hippocampus [78], decreasing the expression of SHH, GLI, PTCH, and SMO proteins [80]. The modulation of proteins of this pathway may lead to an antidepressant effect in models of chronic unpredictable stress [80]. Our bioinformatic analyses pointed to PTCH1 as a potential vitamin D target, with affinity values of −11.570 kcal/mol, similar to VDR values. The relationship between the SHH signaling pathway and depression and anxiety has not been established, but SHH may regulate hippocampal neurogenesis in adults [80]. Thus, more studies are needed to validate PTCH1 as a potential target of calcitriol responsible for its antidepressant and anxiolytic effects.

Interestingly, molecular docking analysis showed that calcitriol (−9.275 kcal/mol) has a higher binding energy for the OPRL1 receptor (NOP receptor) than its known ligand, DGV (−8.291 kcal/mol). To date, to our knowledge, the influence of calcitriol on this receptor has not been investigated, so this is a new potential pharmacological target for vitamin D that could be explored in future studies. Similar to the other opioid receptors, the NOP receptor is coupled to G protein. The binding of the agonist nociceptin/orphanin FQ (N/OFQ) to this receptor induces Gi/o activation with the consequent inhibition of the enzyme adenylate cyclase, which, in turn, leads to reduced concentrations of cyclic adenosine monophosphate and decreased activity of protein kinase A. The Gβγ subunit released upon the agonist binding to the receptor causes inhibition of presynaptic calcium channels (Cav2.1., Cav2.2 and Cav2.3) and activation of the G protein-mediated inward rectifier potassium channel (Kir3). The Gβγ subunit also acts by regulating several intracellular signaling pathways, activating phospholipases and protein kinases, including extracellular signal-regulated kinases 1 and 2 (ERK1/2), p38 mitogen-activated protein kinase (MAPK), and N-terminal c-Jun kinase (JNK) [81,82]. Therefore, the activation of these receptors is related to the modulation of neurotransmitter release and gene transcription [83,84]. In addition, the activation of the NOP receptor can induce NF-κB activation [81,85], and blocking NOP receptor signaling could be a strategy for treating inflammatory diseases [86]. In contrast, treatment with N/OFQ is able to inhibit the synthesis of pro-inflammatory cytokines (IL)-6, IL-1β, and tumor necrosis factor alpha (TNF-α) in the spinal cord and astrocytes [87]. Therefore, it seems that activation of NOP receptors can result in anti- or pro-inflammatory responses [84].

Regarding the role of the NOP receptor in psychiatric disorders, it has been reported that agonists of this receptor have beneficial effects on anxiety [88,89], while antagonists act similarly to antidepressants [90,91,92]. Indeed, treatment with NOP receptor antagonists (UFP-101 and/or SB-612111) attenuates the depressive-like behaviors induced by the chronic mild stress and learned helplessness models in rodents [93,94]. Furthermore, the lipopolysaccharide (LPS) model failed to induce depressive-like behavior in NOP receptor knockout mice (NOP -/-) or mice treated with these antagonists [95]. On the other hand, knockout mice for the NOP receptor exhibited anxiety-like behavior in the elevated plus maze and light–dark box tests, suggesting that the activation of these receptors plays a critical role for anxiolytic effects [96].

Promising results were also obtained when we evaluated the binding energy of calcitriol to phosphodiesterases (PDEs). In relation to PDE5A, calcitriol (−9.230 kcal/mol) had a lower binding energy than the control 5GP (−6.430 kcal/mol). A similar profile occurred in relation to PDE3A, since calcitriol had a binding energy of −8.740 kcal/mol, while the X5M control generated a binding energy of −7.975 kcal/mol. Phosphodiesterases are enzymes that hydrolyze the 3′ phosphate bond of the intracellular second messengers cAMP and cGMP to generate 5′ AMP and 5′ GMP, respectively [97]. These enzymes are classified into 11 families (PDE1-PDE11), each with one to four subtypes [98]. Specifically, PDE5 hydrolyzes cGMP, while PDE3 acts on both cAMP and cGMP [99]. PDEs are present in the brain, and the inhibition of PDEs increases the levels of cAMP and/or cGMP that favors the activation of neurogenic and antioxidant pathways, while inhibiting inflammatory pathways regulated by NF-κB [100,101]. These mechanisms may be related to the beneficial effects of these inhibitors in psychiatric disorders [102]. Particularly, treatment with sildenafil, a PDE5A inhibitor, elicited antidepressant-like effects in models of chronic unpredictable mild stress and LPS [98,103]. In addition, inhibition of this phosphodiesterase also induces anxiolytic-like effects in the elevated plus-maze test [104]. Although there are a limited number of studies, antidepressant [105] and anxiolytic [106] effects have also been observed with cilostazol, a PDE3A inhibitor that has been shown to reduce the decline of cognitive function in patients with Alzheimer’s disease [107]. Although PDE inhibitors are promising therapeutic drugs for neurological diseases, some of them may cause adverse effects. For example, cilostazol may cause headache, diarrhea, abnormal stools, and irregular heart rate and palpitations that limit its use for patients with heart diseases [108,109]. Therefore, considering the therapeutic potential of PDE inhibitors for neurological disorders, including depression and anxiety disorders, calcitriol should be further investigated regarding its ability to inhibit these enzymes.

This study has some limitations. Considering that we conducted a theoretical computational study based on a data mining approach, pharmacological experiments should be performed to better understand the effects of calcitriol on each target protein and how these effects induce an antidepressant-like and/or anxiolytic response. In addition, data on calcitriol as well as psychiatric disorders (MDD and anxiety) were collected through databases, which need to be updated regularly to maintain data accuracy. Another limitation is that molecular docking analyses verify the interaction between the target and the protein through the binding energy value but do not allow per se inferences about the consequences of the interaction. Therefore, future studies using molecular dynamics would be interesting to reinforce our findings.

Considering that our study suggests novel and underexplored proteins as potential molecular targets for the antidepressant and anxiolytic properties of calcitriol, further in silico, in vitro, and in vivo experiments with animal models of depression and anxiety as well as clinical trials are needed to validate these targets. Indeed, future information obtained from human participants may contribute to ascertain the role of the molecular targets proposed herein.

## 4. Materials and Methods

### 4.1. Prediction of Gene Pharmacological Potentials Modulated by Calcitriol in Depression and Anxiety

Initially, the isomeric form of calcitriol was obtained by Puchem (CID 5280453) [110]. After obtaining the molecular structure, the genes modulated by calcitriol were checked using the SwissTargetPrediction software (Swiss Bioinformatics Institute, Lausanne, Switzerland) [111]. By searching for the terms “depression” and “anxiety” in the GeneCards database (Weizmann Institute of Science, Rehovot, Israel), the genes associated with depression and anxiety were obtained. Each search was filtered by the term “Homo sapiens” [112]. The searches were carried out on 19 September 2023. Subsequently, the target genes commonly expressed in depression and anxiety and the calcitriol-related targets were crossed with the Venn map (Venny v.2.1.0-Centro Nacional de Biotecnología, Madrid, Spain).

### 4.2. Gene Interaction Network Construction and Analysis

A pharmacology network was built for each intersection of gene targets in GeneMANIA (University of Toronto, Toronto, Canada). The searches were filtered by the term “Homo sapiens” and the default settings were maintained [113]. The standard GeneMANIA search resulted in 20 more genes than those originally found. The relationship between each node or gene can be of different types: co-expression, co-localization, shared protein domains, physical interactions, genetic interactions, pathways, or predictions. Co-expression means that the gene expression data of the two genes are similar under the same conditions. Co-localization means that the two genes are expressed in the same tissue or that their gene products are expressed in the same cellular compartment. Shared protein domains mean that the two genes have the same protein domain. Physical interaction means that the two gene products physically interact with each other. Genetic interactions occur when two genes share a functional relationship. Pathway means that two genes code for proteins that participate in the same pathway. Predictions means that the relationships between genes are predicted computationally, mainly by orthology. Finally, the networks obtained were improved in Cytoscape V.3.9.1 [114], in which topological analyses were conducted. These analyses allowed for the identification of the most relevant genes in each intersection, using criteria such as degree centrality (DC), betweenness centrality (BC), and closeness centrality (CC). Notably, a certain cut-off value was used in each network to select and keep the most important genes [115], which were selected for the molecular docking analyses [116].

### 4.3. Network Enrichment Analysis

For each network built in Cytoscape, GO and KEGG enrichment analyses were carried out using the ShinyGO v.0.76.3 program (South Dakota State University, Brookings, Washington, DC, USA) [117]. The terms “biological process” (BP), “molecular function” (MF), and “cellular composition” (CC) were selected to perform the GO analyses. In addition, “Homo sapiens”, false discovery rate (FDR) < 0.05, and the 20 most important pathways were used as filters. The most relevant GO analyses or KEGG biochemical pathways were selected based on the FDR values (lower values indicate greater relevance), as done previously [116].

### 4.4. Molecular Docking Simulations

The prediction of molecular interactions between calcitriol and potential protein targets was verified using the DockThor 2.0 software (LNCC, Petrópolis, Brazil) [116,118]. The Protein Data Bank (RCSB) was used to obtain the X-ray diffraction or electron microscopy structures of the main pharmacological targets. AlphaFold Protein Structure Database was used to obtain structural models of proteins whose structure was not available in the PDB. Among the criteria for selecting protein structures, those with the highest quality (resolution < 3Å, lowest Ramachandran outliers, and R-value free) were selected. When available, structures with a holo form (with ligands interacting at the target site) were also prioritized. The structures obtained were then saved in PDB format. Using Chimera 1.14 0 software (UCSF, San Francisco, CA, USA), all the ligands were subjected to geometric optimization [119]. Subsequently, changes were carried out on the target proteins, including the removal of water molecules and ions, the addition of hydrogens, and the assignment of Gasteiger charges. Ligands/antagonists/agonists present in the binding site and allosteric site, as well as chains or parts of chains, were removed from some structures to optimize the docking analysis [116]. The structures and binding sites used are described in the Table 3, Table 4 and Table 5. Proteins whose docking scores were equal to or less than −11 Kcal/mol were selected as biological targets for calcitriol. We also identified as possible targets those proteins whose calcitriol docking score was lower than that of the positive control. In addition, the proteins selected according to these criteria were observed with the poses of the ligands in Chimera 1.14 and analyzed for intermolecular interactions using Ligplot+ + 2.2.5 graphical interface.

### 4.5. Bioinformatic Validation 

To perform a bioinformatic validation, we performed an independent analysis by building a protein–protein interaction (PPI) network with the genes associated with depression downloaded from the DisGeNET database (Mental Depression; CUI: C0011570) and assembling a network in the STRING database (selecting organism Homo sapiens, with a confidence score > 0.7 and no more than 10 interactions in the first and second shells) [120,121]. In addition, a second network was assembled in STRING with the top 10 molecular targets of calcitriol, which are VDR, CB1, PDE5A, PDE3A, CB2, SMO, PTCH1, OPRL1, HRH3, and NR1H3. The two networks were joined in Cytoscape, and the topological parameters were analyzed.

## 5. Conclusions

Our study showed that vitamin D has a polypharmacological profile and may interact with many targets. Among the targets found were VDR (which was already expected) and other novel proteins, namely, the histamine H3 receptor (H3R), the endocannabinoid receptors 1 and 2 (CB1 and CB2), nuclear receptor NR1H3, PTCH1, opioid receptor NOP, and phosphodiesterase enzymes PDE3A and PDE5A. The integrated bioinformatic approach made it possible to select the most promising targets involved in the antidepressant and anxiolytic mechanisms of action of calcitriol, paving the way for future studies that may confirm the relevance of the molecular targets for the antidepressant and anxiolytic properties of vitamin D.

## Figures and Tables

**Table 1:** The 74 genes overlapping calcitriol, depression, and anxiety.

Gene Symbol	Codified Proteins
ABCB1	ATP Binding Cassette Subfamily B Member 1
ABL1	ABL Proto-Oncogene 1, Non-Receptor Tyrosine Kinase
AKT1	AKT Serine/Threonine Kinase 1
AKT2	AKT Serine/Threonine Kinase 2
ALK	ALK Receptor Tyrosine Kinase
AR	Androgen Receptor
CA1	Carbonic Anhydrase 1
CA2	Carbonic Anhydrase 2
CCKBR	Cholecystokinin B Receptor
CCR1	C-C Motif Chemokine Receptor 1
CDC25A	Cell Division Cycle 25A
CHEK1	Checkpoint Kinase 1
CHUK	Component Of Inhibitor Of Nuclear Factor Kappa B Kinase Complex
CNR1	Cannabinoid Receptor 1
CNR2	Cannabinoid Receptor 2
CSF1R	Colony Stimulating Factor 1 Receptor
CSK	C-Terminal Src Kinase
CYP19A1	Cytochrome P450 Family 19 Subfamily A Member 1
CYP24A1	Cytochrome P450 Family 24 Subfamily A Member 1
CYP27B1	Cytochrome P450 Family 27 Subfamily B Member 1
DPP4	Dipeptidyl Peptidase 4
DRD3	Dopamine Receptor D3
EPHX2	Epoxide Hydrolase 2
ESR1	Estrogen Receptor 1
ESR2	Estrogen Receptor 2
EZH2	Enhancer Of Zeste 2 Polycomb Repressive Complex 2 Subunit
FAAH	Fatty Acid Amide Hydrolase
FGFR1	Fibroblast Growth Factor Receptor 1
GC	GC Vitamin D Binding Protein
GHSR	Growth Hormone Secretagogue Receptor
GLRA1	Glycine Receptor Alpha 1
GSK3B	Glycogen Synthase Kinase 3 Beta
HCRTR1	Hypocretin Receptor 1
HCRTR2	Hypocretin Receptor 2
HMGCR	3-Hydroxy-3-Methylglutaryl-CoA Reductase
HRH3	Histamine Receptor H3
HSD11B2	Hydroxysteroid 11-Beta Dehydrogenase 2
HTR2A	5-Hydroxytryptamine Receptor 2A
IKBKB	Inhibitor Of Nuclear Factor Kappa B Kinase Subunit Beta
INSR	Insulin Receptor
JAK1	Janus Kinase 1
JAK2	Janus Kinase 2
KCNH2	Potassium Voltage-Gated Channel Subfamily H Member 2
KDR	Kinase Insert Domain Receptor
LIMK2	LIM Domain Kinase 2
MAP2	Microtubule Associated Protein 2
MAP3K14	Mitogen-Activated Protein Kinase Kinase Kinase 14
MCHR1	Melanin Concentrating Hormone Receptor 1
MDM2	MDM2 Proto-Oncogene
MET	MET Proto-Oncogene, Receptor Tyrosine Kinase
MTOR	Mechanistic Target Of Rapamycin Kinase
NR1H3	Nuclear Receptor Subfamily 1 Group H Member 3
NR1I2	Nuclear Receptor Subfamily 1 Group I Member 2
OPRD1	Opioid Receptor Delta 1
OPRK1	Opioid Receptor Kappa 1
OPRM1	Opioid Receptor Mu 1
P2RX3	Purinergic Receptor P2X 3
PDE10A	Phosphodiesterase 10A
PDE3A	Phosphodiesterase 3A
PDE3B	Phosphodiesterase 3B
PIK3CA	Phosphatidylinositol-4,5-Bisphosphate 3-Kinase Catalytic Subunit Alpha
PRKCB	Protein Kinase C Beta
PRKG1	Protein Kinase CGMP-Dependent 1
PTAFR	Platelet Activating Factor Receptor
REN	Renin
SCN9A	Sodium Voltage-Gated Channel Alpha Subunit 9
SHH	Sonic Hedgehog Signaling Molecule
SLC6A2	Solute Carrier Family 6 Member 2
SLC6A3	Solute Carrier Family 6 Member 3
TACR1	Tachykinin Receptor 1
TACR2	Tachykinin Receptor 2
TBXA2R	Thromboxane A2 Receptor
TGFBR1	Transforming Growth Factor Beta Receptor 1
VDR	Vitamin D Receptor

**Table 2:** Genes overlapping calcitriol, depression, and anxiety obtained in the filtered network.

Gene Symbol	Codified Proteins
ABL1	ABL proto-oncogene 1, non-receptor tyrosine kinase
AKT1	AKT serine/threonine kinase 1
AKT2	AKT serine/threonine kinase 2
ALK	ALK receptor tyrosine kinase
AR	Androgen receptor
CA3	Carbonic anhydrase 3
CAMK1G	Calcium/calmodulin-dependent protein kinase IG
CBL	Cbl proto-oncogene
CCKBR	Cholecystokinin B receptor
CCR1	C-C motif chemokine receptor 1
CHEK1	Checkpoint kinase 1
CHUK	Component of inhibitor of nuclear factor kappa B kinase complex
CNR1	Cannabinoid receptor 1
CNR2	Cannabinoid receptor 2
CSF1R	Colony stimulating factor 1 receptor
CSK	C-terminal Src kinase
CYP19A1	Cytochrome P450 family 19 subfamily A member 1
DRD3	Dopamine receptor D3
ESR1	Estrogen receptor
EZH2	Enhancer of zeste 2 polycomb repressive complex 2 subunit
FGFR1	Fibroblast growth factor receptor 1
GC	GC vitamin D binding protein
GHSR	Growth hormone secretagogue receptor
GSK3B	Glycogen synthase kinase 3 beta
HCRTR1	Hypocretin receptor 1
HCRTR2	Hypocretin receptor 2
HRH3	Histamine receptor H3
HTR2A	5-hydroxytryptamine receptor 2A
IHH	Indian hedgehog signaling molecule
IKBKB	Inhibitor of nuclear factor kappa B kinase subunit beta
INSR	Insulin receptor
JAK1	Janus kinase 1
KDR	Kinase insert domain receptor
KSR1	Kinase suppressor of ras 1
MAP3K14	Mitogen-activated protein kinase kinase kinase 14
MAPT	Microtubule associated protein tau
MDM2	MDM2 proto-oncogene
MET	MET proto-oncogene, receptor tyrosine kinase
MTOR	Mechanistic target of rapamycin kinase
NR1H3	Nuclear receptor subfamily 1 group H member 3
OPRD1	Opioid receptor delta 1
OPRK1	Opioid receptor kappa 1
OPRL1	Opioid related nociceptin receptor 1
OPRM1	Opioid receptor mu 1
PDE3A	Phosphodiesterase 3A
PDE5A	Phosphodiesterase 5A
PIK3CA	Phosphatidylinositol-4,5-bisphosphate 3-kinase catalytic subunit alpha
PIK3R1	Phosphoinositide-3-kinase regulatory subunit 1
PRKCA	Protein kinase C alpha
PRKCB	Protein kinase C beta
PRKG1	Protein kinase cGMP-dependent 1
PTAFR	Platelet-activating factor receptor
PTCH1	Patched 1
REN	Renin
SHH	Sonic hedgehog signaling molecule
TACR1	Tachykinin receptor 1
TACR2	Tachykinin receptor 2
TGFBR1	Transforming growth factor beta receptor 1
VDR	Vitamin D receptor

**Table 3:** Docking scores of main targets of calcitriol related to depression and anxiety.

Gene Symbol	PDB ID	Ligand ID (Binding Site)	Positive Control (Ligand ID) Docking Score (kcal/mol)	Calcitriol Docking Score (kcal/mol)
VDR	1DB1	VDX	−12.116	−12.116
HRH3	7F61	1IB	−10.509	−12.077
NR1H3	3IPQ	965	−13.750	−11.927
CNR1	7WV9	9GF	−11.133	−11.810
PTCH1	6RTW	Y01	−11.908	−11.570
CNR2	6PT0	WI5	−11.502	−11.333
HTR2A	6A93	8NU	−11.220	−10.639
CYP19A1	3ST9	ASD	−9.048	−10.024
HCRTR2	4S0V	SUV	−10.610	−9.920
TACR2	7XWO	α-helix	N.A.	−9.903
MTOR	4DRH	RAP	−14.977	−9.871
GHSR	7NA8	1KD	−10.476	−9.803
CCKBR	7XOW	gastrine	N.A.	−9.674
ESR1	1A52	EST	−9.860	−9.622
TACR1	6E59	L76	−10.985	−9.607
HCRTR1	4ZJ8	SUV	−10.690	−9.450
PRKCA	3IW4	LW4	−10.166	−9.324
OPRL1	5DHG	DGV	−8.291	−9.275
ABL1	1OPL	P16	−10.757	−9.245
PDE5A	1T9S	5GP	−6.430	−9.230
TGFBR1	1PY5	PY1	−9.168	−9.230
REN	1BIL	0IU	−11.031	−9.219
PRKCB	2I0E	PDS	−9.299	−9.160
CHUK	5EBZ	5TL	−8.347	−9.137
OPRD1	6PT2	KGCHM07 (peptide)	N.A.	−9.128
CCR1	7VL9	CLR	−8.753	−9.090
DRD3	8IRT	R5F	−10.321	−9.054
KDR	1Y6A	AAZ	−9.908	−8.914
INSR	1GAG	112	−7.707	−8.901
CSK	1BYG	STU	−9.931	−8.810
CAMK1G	2JAM	J60	−9.240	−8.754
PDE3A	7KWE	X5M	−7.975	−8.740
OPRM1	8EF5	7V7	−9.767	−8.639
CNR2	6PT0	CLR	−8.361	−8.625
GSK3B	6B8J	65C	−9.243	−8.612
PIK3CA	3ZIM	KKR	−10.432	−8.518
OPRK1	4DJH	JDC	−11.363	−8.457
EZH2	5HYN	SAH	−8.392	−8.394
FGFR1	3DPK	8C5	−9.543	−8.309
AKT2	1GZK	α-helix	N.A.	−8.157
CNR1	7WV9	7IC	−8.213	−8.038
GC	1J78	VDY	−8.621	−8.034
PTAFR	P25105 *	α-helix	N.A.	−8.020
MDM2	1RV1	IMZ	−9.773	−7.993
CSF1R	3BEA	IXH	−10.084	−7.959
KSR1	7JUY	ANP	−6.572	−7.950
MAPT	7NRS	center of protein	N.A.	−7.891
ALK	2XP2	VGH	−8.907	−7.871
IHH	3K7I	α-helix	N.A.	−7.851
AR	1T5Z	DHT	−10.094	−7.746
IKBKB	4KIK	KSA	−10.662	−7.730
JAK1	4E4L	0NH	−8.583	−7.342
AKT1	2UVM	GVF	−7.453	−7.268
PIK3R	1H9O	PTR	−6.556	−7.267
SHH	6PJV	GOL	N.A.	−7.187
MAP3K14	4DN5	AGS	−6.658	−7.130
CHEK1	1ZLT	HYM	−7.915	−7.127
CA3	3UYQ	α-helix	N.A.	−6.963

N.A. Positive control not available. * AlphaFold model.

**Table 4:** Docking scores of main targets of calcitriol related to depression.

Gene Symbol	PDB ID	Ligand ID (Binding Site)	Positive Control (Ligand ID) Docking Score (kcal/mol)	Calcitriol Docking Score (kcal/mol)
SMO	4QIM	ANTAXV(A8T)	−11.921	−10.995
HTR1D	7E32	SRO	−7.735	−10.563
EBP	6OHU	CTX	−11.329	−10.509
GPBAR1	7CFM	P395 (FWX)	−9.368	−10.253
S1PR2	7T6B	S1P	−8.881	−9.907
S1PR1	7E04	BAF312 (J8C)	−10.775	−9.701
ADAMTS5	3B8Z	294	−10.223	−8.761
GSK3A	7SXG	BIO8546(D1E)	−8.959	−8.426
ACAN	4MD4	α-helix	N.A.	−8.421
ADAMTS4	2RJP	886	−10.875	−8.416
BRSK2	Q8IWQ3 *	α-helix	N.A.	−8.348
S1PR3	7EW2	EFTY720(J89)	−9.144	−8.273
STAT6	4Y5W	α-helix	N.A.	−8.244
HTR1D	7E32	CLR	−8.750	−8.153
EPHB4	2VWU	7 × 1	−10.345	−8.062
JAK3	3ZC6	VFC	−9.065	−7.872
TTK	2X9E	NMS-P715 (SVE)	−10.407	−7.823
EPHB3	5L6O	6P6	−8.676	−7.622
WEE1	3BI6	PD352396 (396)	−10.779	−7.510
AKT3	2 × 18	EPE	−6.106	−7.379
PRKD3	2D9Z	α-helix	N.A.	−7.125
PTPN1	1BZC	TPI	−7.353	−6.496

N.A. Positive control not available. * AlphaFold model.

**Table 5:** Docking scores of the main targets of calcitriol related to anxiety.

Gene Symbol	PDB ID	Ligand ID (Binding Site)	Positive Control (Ligand ID) Docking Score (kcal/mol)	Calcitriol Docking Score (kcal/mol)
AURKB	4AF3	VX6	−10.203	−10.268
HSP90B	3NMQ	EC44/7PP	−9.434	−10.195
AURKC	6GR9	VX6	−7.638	−9.892
INCENP	6GR8	α-helix	N.A.	−7.920
BIRC5	2QFA	MES	−6.717	−6.374

N.A. Positive control not available.

**Table 6:** PPI network topological parameters of calcitriol targets in depression.

Gene/ Protein Symbol	Degree	Betweenness Centrality	Closeness Centrality	Clustering Coefficient
CNR1/CB1	27	0.0019	0.3379	0.2821
CNR2/CB2	7	0.0017	0.2923	0.4286
PDE3A	8	9.82 × 10^8^	0.2651	0.7857
PDE5A	9	1.15 × 10^10^	0.2652	0.8056
PTCH1	3	0.0000	0.2173	1.0000
SMO	4	0.0049	0.2775	0.5000

## Data Availability

The original contributions presented in the study are included in the article/Supplementary Material, further inquiries can be directed to the corresponding author.

## Supplementary Materials

The following supporting information can be downloaded at: https://www.mdpi.com/article/10.3390/ph17070893/s1, Table S1: Parameters of docking sites for targets shared by calcitriol, depression and anxiety; Table S2: Parameters of docking sites for targets shared by calcitriol and depression; Table S3: Parameters of docking sites for targets shared by calcitriol and anxiety.
