# Supplementary material for: Putative Pharmacological Depression and Anxiety-Related Targets of Calcitriol Explored by Network Pharmacology and Molecular Docking
Source: Pharmaceuticals (Basel). 2024 Jul 5;17(7):893. doi: 10.3390/ph17070893 (PMC11280388; doi:10.3390/ph17070893)
Supplement: Supplementary file 1 [file pharmaceuticals-17-00893-s001.zip › pharmaceuticals-3073213-supplementary.pdf]

Supplementary Data.

Table S1. Parameters of docking sites for targets shared by calcitriol, depression and anxiety.

Gene Symbol	PDB ID	Ligand ID (Binding Site)	Gridbox (Center)	Gridbox (Size)
VDR	1DB1	VDX	10 24 35	24 24 24
HRH3	7F61	1IB	-20 50 0	24 24 24
NR1H3	3IPQ	965	42 18 -5	24 24 24
CNR1	7WV9	9GF	136 102 93	24 24 24
PTCH1	6RTW	Y01	-15 7,5 -30	24 24 24
CNR2	6PT0	WI5	100 110 125	24 24 24
HTR2A	6A93	8NU	15 0 60	24 24 24
CYP19A1	3ST9	ASD	87 55 45	24 24 24
HCRTR2	4S0V	SUV	50 9 55	24 24 24
TACR2	7XWO	α-helix	115 100 145	24 24 24
MTOR	4DRH	RAP	-5 20 -2	24 24 24
GHSR	7NA8	1KD	125 135 93	24 24 24
CCKBR	7XOW	gastrine	93 115 67	24 24 24
ESR1	1A52	EST	106 15 95	24 24 24
TACR1	6E59	L76	-15 55 55	24 24 24
HCRTR1	4ZJ8	SUV	-8 0 -55	24 24 24
PRKCA	3IW4	LW4	6 30 52	24 24 24
OPRL1	5DHG	DGV	-8 30 -2	24 24 24
ABL1	1OPL	P16	20 52 50	24 24 24
PDE5A	1T9S	5GP	40 46 1	24 24 24
TGFBR1	1PY5	PY1	5 10 6	24 24 24
REN	1BIL	0IU	37 60 43	24 24 24
PRKCB	2I0E	PDS	40 55 35	24 24 24
CHUK	5EBZ	5TL	86 65 2	24 24 24
OPRD1	6PT2	KGCHM07 (peptide)	-20 40 -28	24 24 24
CCR1	7VL9	CLR	125 125 130	24 24 24
DRD3	8IRT	R5F	108 130 120	24 24 24
KDR	1Y6A	AAZ	0 30 18	24 24 24
INSR	1GAG	112	-22 28 9	24 24 24
CSK	1BYG	STU	26 45 13	24 24 24
CAMK1G	2JAM	J60	15 3 13	24 24 24
PDE3A	7KWE	X5M	-18 -20 43	24 24 24
OPRM1	8EF5	7V7	100 106 127	24 24 24
CNR2	6PT0	CLR	90 95 135	24 24 24
GSK3B	6B8J	65C	-2 42 19	24 24 24
PIK3CA	3ZIM	KKR	-23 12 28	24 24 24
OPRK1	4DJH	JDC	3 -23 60	24 24 24
EZH2	5HYN	SAH	80 -98 2	24 24 24
FGFR1	3DPK	8C5	5 27 8	24 24 24
AKT2	1GZK	α-helix	-20 120 -70	24 24 24
CNR1	7WV9	7IC	125 90 100	24 24 24
GC	1J78	VDY	-21 -60 47	24 24 24
PTAFR	AF-P25105*	α-helix	12 3 -12	24 24 24
MDM2	1RV1	IMZ	10 10 14	24 24 24
CSF1R	3BEA	IXH	5 30 8	24 24 24
KSR1	7JUY	ANP	-5 65 -3	24 24 24
MAPT	7NRS	center of protein	112 130 132	24 24 24
ALK	2XP2	VGH	29 45 9	24 24 24
IHH	3K7I	α-helix	-5 -25 3	24 24 24
AR	1T5Z	DHT	0 60 3	24 24 24
IKBKB	4KIK	KSA	48 30 -58	24 24 24
JAK1	4E4L	0NH	-12 28 0	24 24 24
AKT1	2UVM	GVF	15 11 15	24 24 24
PIK3R	1H9O	PTR	14 10 20	24 24 24
SHH	6PJV	GOL	-35 47 20	24 24 24
MAP3K14	4DN5	AGS	-10 30 -5	24 24 24
CHEK1	1ZLT	HYM	15 0 13	24 24 24
CA3	3UYQ	α-helix	60 12 30	24 24 24

* AlphaFold model

Table S2. Parameters of docking sites for targets shared by calcitriol and depression.

Gene Symbol	PDB ID	Ligand ID (Binding Site)	Gridbox (Center)	Gridbox (Size)
SMO	4QIM	ANTAXV(A8T)	14 -12 -23	24 24 24
HTR1D	7E32	SRO	100 121 112	24 24 24
EBP	6OHU	CTX	-15 10 26	24 24 24
GPBAR1	7CFM	P395 (FWX)	99 120 113	24 24 24
S1PR2	7T6B	S1P	112 145 138	24 24 24
S1PR1	7E04	BAF312 (J8C)	129 137 169	24 24 24
ADAMTS5	3B8Z	294	13 3 -5	24 24 24
GSK3A	7SXG	BIO8546(D1E)	-15 -15 17	24 24 24
ACAN	4MD4	α-helix	55 27 -6	24 24 24
ADAMTS4	2RJP	886	64 0 15	24 24 24
BRSK2	AF-Q8IWQ3*	α-helix	-20 10 0	24 24 24
S1PR3	7EW2	EFTY720(J89)	118 102 78	24 24 24
STAT6	4Y5W	α-helix	24 15 18	24 24 24
HTR1D	7E32	CLR	83 120 96	24 24 24
EPHB4	2VWU	7X1	12 9 12	24 24 24
JAK3	3ZC6	VFC	12 -2 44	24 24 24
TTK	2X9E	NMS-P715 (SVE)	-8 20 -1	24 24 24
EPHB3	5L6O	6P6	15 5 -5	24 24 24
WEE1	3BI6	PD352396 (396)	5 54 24	24 24 24
AKT3	2X18	EPE	24 66 -20	24 24 24
PRKD3	2D9Z	α-helix	-15 1 0	24 24 24
PTPN1	1BZC	TPI	-20 54 16	24 24 24

* AlphaFold model

Table S3. Parameters of docking sites for targets shared by calcitriol and anxiety.

Gene Symbol	PDB ID	Ligand ID (Binding Site)	Gridbox (Center)	Gridbox (Size)
AURKB	4AF3	VX6	22 -20 -10	24 24 24
HSP90B	3NMQ	EC44/7PP	30 10 8	24 24 24
AURKC	6GR9	VX6	30 8 7	24 24 24
INCENP	6GR8	α-helix	40 9 -18	24 24 24
BIRC5	2QFA	MES	45 6 42	24 24 24
